# Late-onset Mitochondrial Encephalomyopathy with Lactic Acidosis and Stroke-like Episodes (MELAS) Syndrome in a 63-year-old Patient

**DOI:** 10.7759/cureus.7862

**Published:** 2020-04-27

**Authors:** Hassan Abdullah, Syed Shah, Humza Husain, Furqan Hassan, Hamza Maqsood

**Affiliations:** 1 Medicine, Nishtar Medical University, Multan, PAK; 2 Neurology, University of Alabama, Birmingham, USA; 3 Medicine, Nishtar Hospital, Nishtar Medical University, Multan, PAK; 4 Internal Medicine, Nishtar Medical University, Multan, PAK

**Keywords:** melas, stroke, myopathy, encephalopathy, lactic acidosis, seizures, mri, mitochondrial dna mutation

## Abstract

Mitochondrial encephalomyopathy with lactic acidosis and stroke-like episodes (MELAS) usually manifests in early life. Clinical hallmarks of the disease are mitochondrial myopathies, encephalopathy with stroke-like episodes, seizures, and lactic acidosis. It rarely manifests in late adulthood. Here we present the case of a 63-year-old female patient who developed recurrent stroke-like symptoms with typical resolving and remitting pattern of findings on imaging. Later on, it was confirmed as a case of MELAS upon genetic analysis.

## Introduction

Mitochondrial encephalomyopathy with lactic acidosis and stroke-like episodes (MELAS) is a maternally inherited multi-systemic disorder caused by mutations of mitochondrial DNA, which leads to a respiratory chain deficiency. It typically manifests in childhood after a normal initial development [1,2]. Signs and symptoms comprise mitochondrial myopathy, encephalopathy with stroke-like episodes, seizures, and/or dementia and lactic acidosis [3,4]. We present the case of a female patient who developed late-onset MELAS at 63 years of age.

## Case presentation

**Figure 1:**
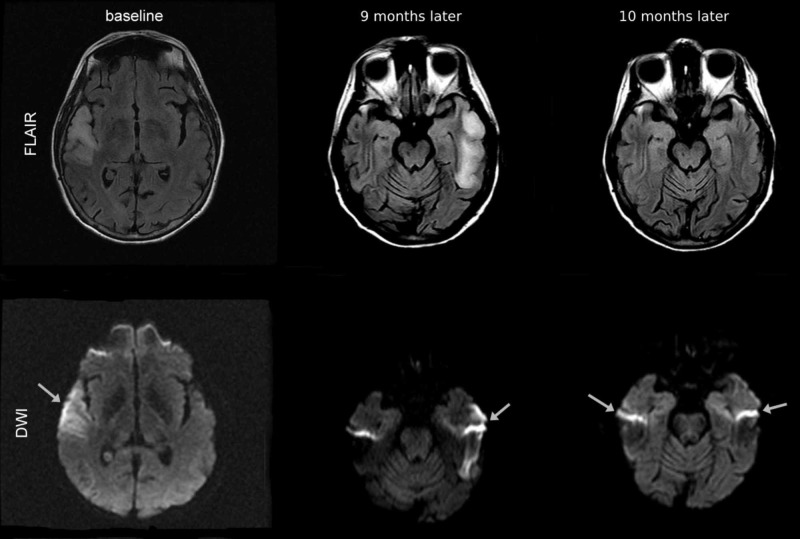
MRI FLAIR and DWI images at first presentation and until 10 months later are presented. FLAIR images demonstrate step-wise progressing FLAIR hyperintense edema not corresponding to a vascular territory. DWI delineates increased signal (T2 shine-through), as shown by the arrows. MRI, magnetic resonance imaging; FLAIR, fluid-attenuated inversion recovery; DWI, diffusion-weighted imaging

A 63-year-old woman got admitted to our hospital with left-sided headache, difficulty in finding words, toothache, leg pain, and odynophagia for one week. On clinical examination, she had left upper extremity ataxia, moderate expressive and receptive aphasia with paraphasic errors, and anomia. Her National Institutes of Health Stroke Scale (NIHSS) score was 3 (minor stroke). She had a medical history of diabetes mellitus, hyperlipidemia, chronic obstructive pulmonary disease (COPD), and transient ischemic strokes. Nine months back, she presented to an outside hospital with similar episodes where her condition was considered for stroke versus inflammatory changes. At that time, she deferred a lumbar puncture (LP) for the inflammatory workup. She had a baseline MRI with right temporal hyperintensity on fluid-attenuated inversion recovery (FLAIR) (Figure 1). There was no diffusion restriction on diffusion-weighted imaging (DWI) because the corresponding apparent diffusion coefficient (ADC) was normal. She had no history of weakness of extremities, numbness, or incoordination. Her childhood and early adulthood were normal, though she developed deafness in her late adulthood. A detailed family history revealed hearing loss at a late age in her mother and a maternal aunt, lung cancer in her father, and a history of stroke in a brother at 57 years of age. The patient has no children (the spouse is infertile). She was on aspirin, Amaryl,® Lantus®, Lipitor®, and Zetia®.

On admission, her vitals were stable, and laboratory testing was normal except for her serum lactate level, which was 2 mmol/L. Computed tomography (CT) showed cortically based hypo-density in the lateral and posterior left temporal lobes that was opposite to her previous scans, where the abnormality was in the right lobe. The findings were nonspecific but could indicate acute infarction or focal encephalitis. CT angiogram showed no relevant stenosis or occlusion of intracranial or extra-cranial arteries. Therefore, further investigations with pre- and post-contrast MRI were recommended.

MRI of the brain without contrast (nine months later from the baseline MRI; Figure 1) showed cortical swelling and increased T2/FLAIR signal involving the left temporal lobe. DWI delineated increased signal (T2 shine-through) without any significant change on ADC, which was thought to represent vasogenic rather than cytotoxic edema. It was likely suggestive of sub-acute infarct similar to the previously seen lesion in the right temporal lobe.

**Figure 2:**
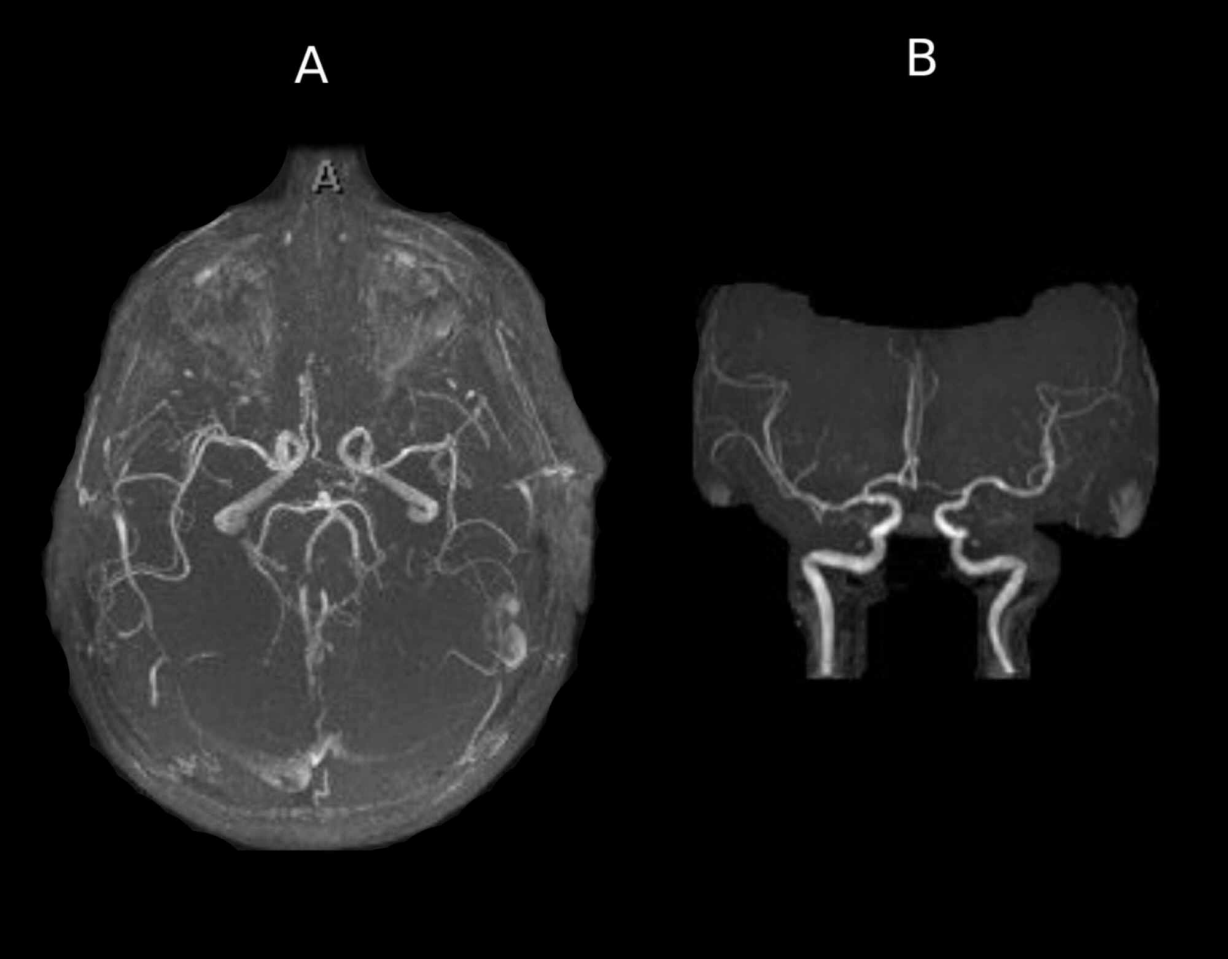
MRA. (A) Axial view. (B) Coronal view. MRA did not reveal any evidence of intracranial arterial stenosis or occlusion. MRA, magnetic resonance angiography

Magnetic resonance angiography (MRA) did not reveal flow-limiting stenosis or any evidence of acute infarct or intracranial hemorrhage (Figure 2).

**Figure 3:**
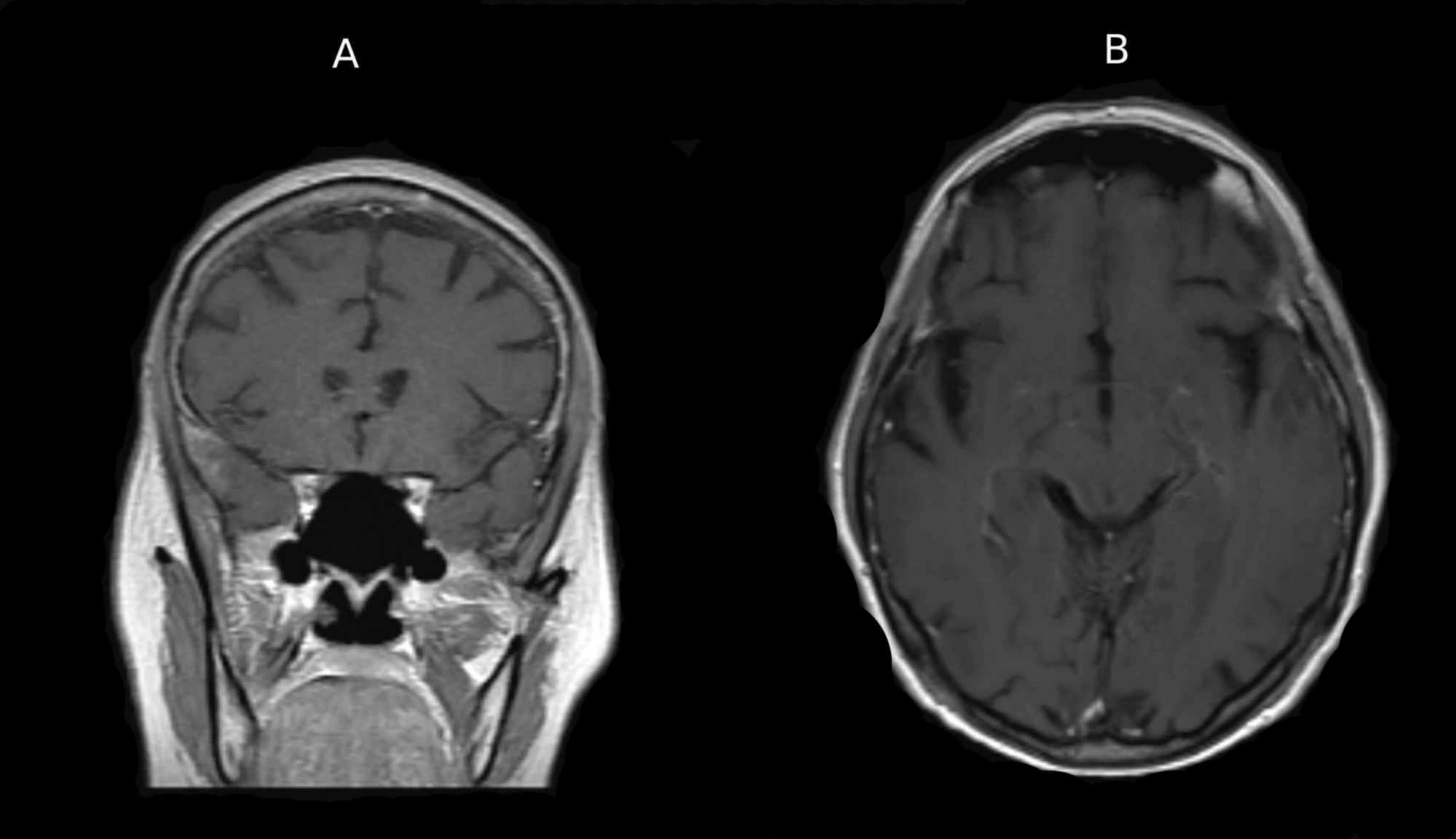
MRI. (A) Coronal view. (B) Axial view. T1-weighted MRI with contrast showing no post-contrast enhancement in the left temporal region in both views. MRI, magnetic resonance imaging

MRI of the brain with contrast (Figure 3) showed no post-contrast enhancement in the region of the left temporal lesion.

Taken in conjunction with the now resolved previous lesion in the right temporal lobe (Figure 1), we considered encephalitis in the differential diagnosis.

The LP was normal with two WBCs, zero RBC, protein 51, and glucose 75, and negative for culture and cryptoantigens.

Transthoracic echocardiography (ECG) revealed no source of any cardiac embolism. ECG monitoring reported no atrial fibrillation. Since long-term EEG (electroencephalogram) monitoring showed no epileptiform discharges, so stopped Keppra®, which was given prophylactically at an outside hospital. At that time, MRI findings were consistent with cerebral edema of an unknown etiology. There was no diffusion restriction to suggest a stroke. Moreover, the absence of any abnormal contrast enhancement and the resolution of the right temporal lobe lesion and subsequently the left temporal lobe edema were decidedly unusual.

Initially, the inpatient team considered posterior reversible encephalopathy syndrome (PRES), but the edema was more cortical and sub-cortical. We equally considered MELAS in our differential diagnosis due to her family history of hearing loss and short stature. Against it, was a late onset of disease. Therefore, for further investigations, the patient was referred to the genetics clinic for gene mutation testing for MELAS. We scheduled a follow-up visit after one month. At the time of discharge, our primary discharge diagnosis was PRES. Aspirin, atorvastatin, calcium-magnesium carbonate, ezetimibe, glimepiride, and insulin were prescribed as outpatient medications. We also recommended speech therapy for her. Her paraneoplastic panel results were pending.

The patient denied any sudden change in vision and speech, lateralized weakness, numbness, or incoordination during that one-month interval upon her follow-up visit. Her NHISS score was 1. Serum lactate and pyruvate levels were in the standard range. The serum paraneoplastic panel including anti-neuronal antibodies turned out to be negative. MRI of the brain without contrast (10 months later from the baseline MRI; Figure 1) showed bilateral temporal polar signal abnormalities that appeared to be chronic. We observed no acute appearing brain edema or mass effect. Diffuse brain atrophy was present. All the infectious workup for herpes and autoimmune encephalitis was negative.

One and a half months after that follow-up, we received the official results of genetic testing. Mitochondrial genetic testing consuming blood as a sample showed heteroplasmic m.3243A>G mitochondrial DNA mutation in the MT-TL1- gene. Hence, our terminal diagnosis was MELAS. We started with a low dose of arginine (1,000-2,000) and CoQ10 at 60 mg daily, and scheduled a follow-up visit at the genetics clinic after one month. During the genetics clinic visit, the consultant recommended increasing the CoQ10 dose, if tolerated, to 300 mg PO daily and arginine to 200 mg PO thrice a day, if tolerated.

The patient is still alive and doing well. She follows on up with our stroke service. Her condition is improving. We added vitamin B12 1 mg daily as it was low. The rest of the medications are the same.

## Discussion

The typical age of clinical presentation in MELAS is highly variable, but the first episode predominantly occurs before the age of 40. The educative value of our case lies in distinguishing findings on MRI, unusual age of presentation, and physical examination (short stature, hearing loss), which finally lead to an accurate diagnosis.

In our patient, the stroke-like episode first occurred at 63 years of age. There are very few case reports or case series on this topic. Indeed, our initial diagnosis was PRES. Castillo published the radiographic findings in eight cases of MELAS. One of the patients was 80 years old and another was 60 years old [5]. The clinical course of the disease, medical history, family history, clinical examination, and distinct MRI findings in our case lead us to the suspicion of MELAS, which was later confirmed by molecular genetic testing. A muscle biopsy was not performed in our case.

MELAS is characterized by high variability of mitochondrial mutation load in a different individual of the same affected family, different organs of the same person, and even in different cells of the same organ, a phenomenon known as heteroplasmy [6]. It might explain different clinical phenotypes with varying ages of presentation and even missed disease in a family history.

**Figure 4:**
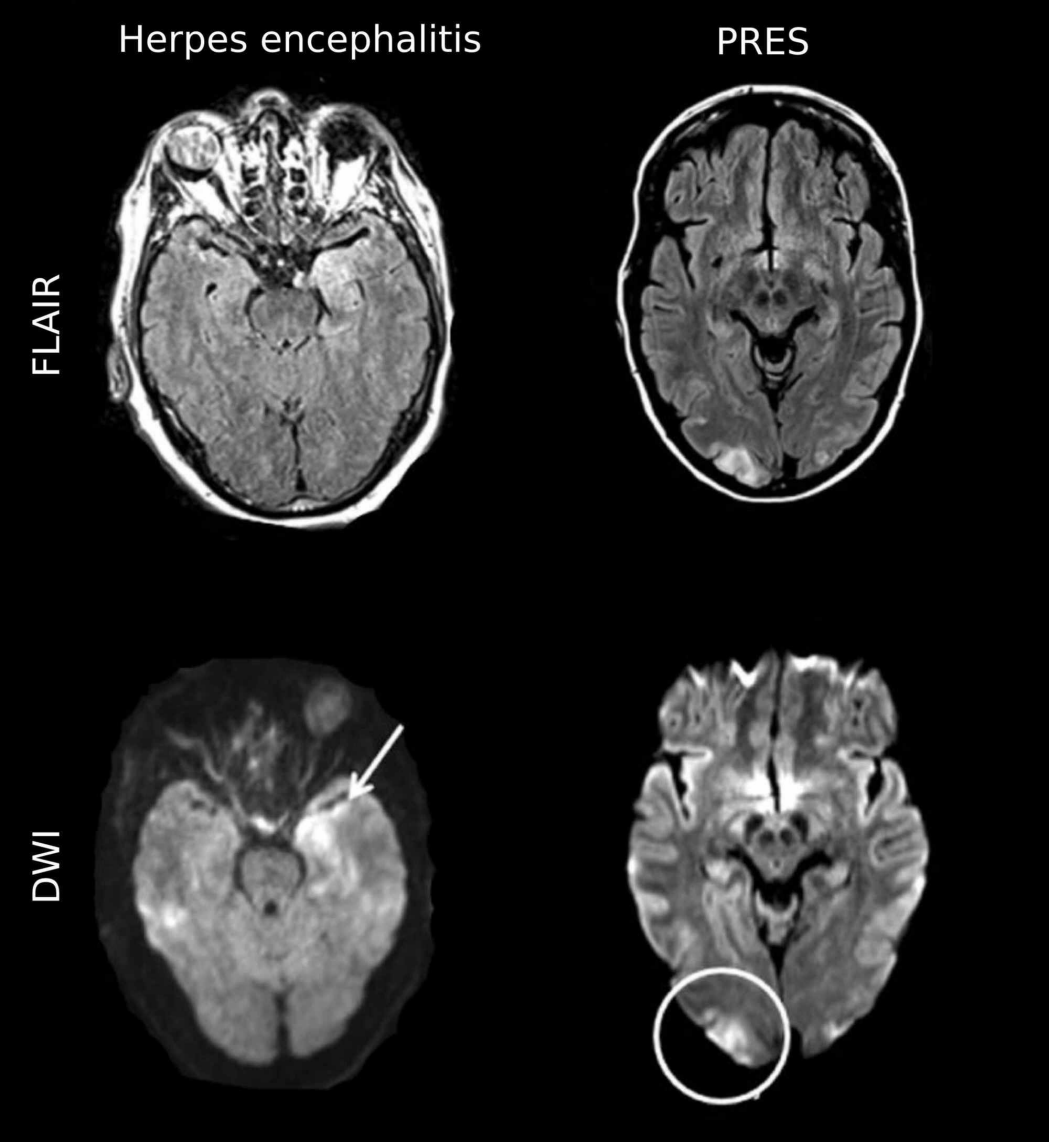
Imaging findings in MELAS differential diagnoses Exemplary FLAIR and DWI images of diseases that can mimic MELAS lesions on MRI, including herpes simplex encephalitis and PRES, are displayed. Herpes simplex encephalitis may mimic many MELAS MRI findings including cortical restricted diffusion (arrow), subcortical vasogenic edema, and local mass effect on FLAIR images. Nevertheless, herpes simplex encephalitis lesions usually affect mesiotemporal areas and spread continuously. PRES lesions are typically located in the occipital and temporal lobes, showing subcortical vasogenic edema (circle) [8]. FLAIR, fluid-attenuated inversion recovery; DWI, diffusion-weighted imaging; MELAS, mitochondrial encephalomyopathy with lactic acidosis and stroke-like episodes; MRI, magnetic resonance imaging; PRES, posterior reversible encephalopathy syndrome

Initially, based on MRI findings, we considered PRES and MELAS in our differential. MELAS lesions are typically localized in the temporoparietal cortex and may progress to the adjacent areas without respecting arterial territories [7]. Various conditions can mimic MELAS on MRI, such as HSV encephalitis and PRES (Figure 4). These two can be differentiated based on typical imaging patterns [8]. Herpes encephalitis can mimic MELAS as it typically affects both cortical and sub-cortical temporal lobes bilaterally [9]. However, the step-wise progression of the lesion is uncommon in herpes encephalitis, and lesions are typically located mesiotemporally [9]. PRES typically shows vasogenic edema in the sub-cortical areas of the occipital and temporal lobes [10]. But in our case, there was cortical involvement as well, which is unusual for PRES.

The best treatment for MELAS to date is limited to a supplementation of CoQ10, L-carnitine, and L-arginine, a non-essential amino acid involved in NO synthesis and endothelial-dependent vascular relaxation, which may explain its benefits particularly in the acute phase of the disease [11].

## Conclusions

Consequently, based on our case study, we conclude that MELAS should be considered in patients with recurrent stroke-like episodes and atypical stroke-like imaging even at the late age of presentation. There should be appropriate radiological and biochemical investigations with ultimate genetic analysis. Otherwise, adult-onset MELAS can go undiagnosed. After diagnoses, a multidisciplinary management of patients with MELAS is required and should involve a geneticist, neurologist, cardiologist, nephrologist, ophthalmologist, and endocrinologist.
